# Gene therapy for hemoglobin disorders - a mini-review

**Published:** 2016

**Authors:** Parul Rai, Punam Malik

**Affiliations:** 1 Cincinnati Children’s Hospital Medical Center, Cincinnati, OH, USA; 2 Department of Pediatrics, University of Cincinnati College of Medicine, Cincinnati, OH 45229, USA

**Keywords:** Gene, Hemoglobinopathies, Sickle cell anemia, Thalassemia, Hemoglobin, Therapy, Review, Disorders

## Abstract

Gene therapy by either gene insertion or editing is an exciting curative therapeutic option for monogenic hemoglobin disorders like sickle cell disease and β-thalassemia. The safety and efficacy of gene transfer techniques has markedly improved with the use of lentivirus vectors. The clinical translation of this technology has met with good success, although key limitations include number of engraftable transduced hematopoietic stem cells and adequate transgene expression that results in complete correction of β0 thalassemia major. This highlights the need to identify and address factors that might be contributing to the in-vivo survival of the transduced hematopoietic stem cells or find means to improve expression from current vectors. In this review, we briefly discuss the gene therapy strategies specific to hemoglobinopathies, the success of the preclinical models and the current status of gene therapy clinical trials.

## Introduction

Sickle cell disease (SCD) and β-thalassemia are autosomal recessive disorders that result in qualitative and quantitative defects in β-globin protein production; and are highly prevalent worldwide, with approximately 7% of the global population estimated to be carriers of hemoglobin gene-variants, and over 330,000 affected infants born annually with SCD alone^1^. Despite improved medical supportive therapies, significant long-term mortality and morbidity associated with hemoglobinopathies remains^2,3^. Hematopoietic stem cell transplant (HSCT) provides the only definitive cure, with a disease free survival exceeding 80% with HLA-matched sibling donor transplants^4,5^. Improvements in management of graft versus host disease (GVHD) and better means of inducing graft tolerance have encouraged the use of an extended donor pool comprising of unrelated donors and umbilical cord blood as the hematopoietic stem cell (HSC) source for patients lacking a matched sibling donor^6^. However, matched-unrelated HSCT for thalassemia have an overall survival of 65% in the high risk patients^7^. In addition, a 5–10% mortality from transplant-conditioning, GVHD and graft failure continues to limit the acceptability of this treatment modality^8^.

Gene therapy, using genetically-modified autologous HSCs is an attractive alternative to allogeneic HSCT, and specifically unrelated-donor HSCT, since it eliminates the need for a matched donor and the risk of GVHD/graft rejection. Successful gene therapy for monogenic immune disorders like chronic granulomatous disease and severe combined immunodeficiency^9–13^ has encouraged development of this technology for hemoglobinopathies. Whereas in immunodeficiency disorders, the genetically modified hematopoietic progenitors and T cells have a selective survival advantage and a tremendous expansion potential, respectively, requiring a minimal (0.1–1%) gene corrected HSC engraftment for sustained correction of lymphoid dysfunction^14^; in hemoglobinopathies, no such survival advantage of genetically modified HSCs/progenitors is present, and selective advantage is limited to the terminal erythroid cells^15^. Post-transplant follow-up studies^16^ and murine models^17,18^ show that a 20% donor chimerism is essential for improving the clinical manifestations in SCD and thalassemia, a level requiring substantial pre-transplant chemotherapy conditioning. Furthermore, in order for globin gene transfer to affect a cure, high level erythroid lineage specific expression is necessary.

Despite these hurdles, improved vector potency and safety have significantly advanced the field, resulting in cures in patients with Hemoglobin E-β-thalassemia and considerable disease amelioration in some patients with β0 thalassemia and SCD. The lessons learnt from these early gene therapy trials suggest that engraftment of sufficient transduced HSC, or their in-vivo selection could play a crucial role to extend the curative capacity of gene therapy.

## Vector development for hemoglobin disorders

Correction of hemoglobin disorders by vector-mediated gene transfer requires utilization of a safe delivery vehicle/vector to efficiently transfer the complex β-transgene cassette to HSCs and result in sustained high expression of the transferred globin gene. The vectors commonly used have been bioengineered from different retroviruses, mainly murine Moloney leukemia virus (retrovirus vectors; RV), HIV-1 (lentivirus vectors; LV) and foamy virus, after removing the genetic elements responsible for their pathogenicity and virulence, and adding the β-globin gene and its locus control region (LCR) elements. Of these, LV have been most successful at correcting hemoglobinopathy animal models, and have resulted in their clinical translation.

The β-globin LCR is a cis-regulatory element composed of five DNAase-1 hypersensitivity sites, four of which are formed in the erythroid cells^19^. When linked to the globin genes LCR leads to position-independent, erythroid lineage-specific enhancement of globin gene expression. The enhancer activity of LCR resides in three of its hypersensitivity sites HS 2, 3 and 4, which contain an array of binding sites for ubiquitous and erythroid specific transcription factors^20^. An intact LCR (5’HS 1–5) is involved in maintaining an open chromatin conformation that is needed for position independent expression of the globin genes. The LCR also results in developmental regulation of globin expression and interacts with the ε, γ and β globin gene promoters in the embryonic, fetal and adult stage, respectively^21^.

### Gamma-retrovirus (RV) vectors

Initial studies looking at RV-mediated human β-globin gene transfer without inclusion of the LCR elements, showed variable and low levels of gene expression (<1% of endogenous β- globin expression)^22^. Following this study, nearly one decade of efforts to develop RV for expressing sufficient globin gene expression were futile^23,24^.

RVs utilizing the enhancer/promoter sequences of the LTR (long terminal repeat) to drive transgene expression of genes other than globin genes, were the first ones to be used in clinical trials. Despite their initial clinical success in gene therapy of immune-deficiencies, concerns about their safety emerged following reports of vector-mediated insertional mutagenesis^9–12^. Integration site analysis revealed that the RVs have a tendency to integrate near cellular promoters, retroviral common integration sites (CIS) and cancer genes, independent of the vector design, and enhance their expression via the LTR promoter/enhancer. The RV vector insertions increase immortalization of primary hematopoietic progenitor cells^25^. While the RV LTR is a strong enhancer and upregulates transgene expression to very high levels compared to relatively weaker enhancers from the HIV LTR and cytomegalovirus^26^, it also simultaneously activates cellular proto- oncogenes flanking insertion sites^27^. Additionally, methylation of the LTR can lead to inactivation of the integrated transgene promoter and prevent long-term transgene expression^28^. The construction of a self-inactivating [SIN] vector design deletes the LTR promoter/enhancer and allows the transgene expression to be driven by internal cellular promoters, reduce LTR enhancer-mediated genotoxicity^27^ and its methylation-induced inactivation^29^. Inclusion of the chicken β-globin hypersensitive site-4 (cHS4) insulator element to the SIN vector further improved its safety by reducing position dependent variablility in gene expression^30^. However the inability of RV to transduce non-diving cells, along with vector instability seen with incorporation of large LCR sequences greatly limited their use in gene therapy for hemoglobin disorders^31^.

### Lentivirus vectors

The interest in LV was generated with the increasing knowledge of the basic structure and properties of HIV-1 virus. HIV-1 can efficiently translocate the intact nuclear membrane and thus, has the ability to transduce non-dividing/quiescent cells and can carry larger expression cassettes. These features enabled the HIV-1 based LVs to be developed for hemoglobinopathies, and efficiently transfer the β-transgene/LCR to HSCs for sustained correction of the hemoglobin defect. The major safety concerns with the use of LVs initially were risk of generating a replication-competent lentivirus (RCL) and insertional mutagenesis. The former risk has been eliminated by removal of HIV regulatory and accessory genes from vector plasmids and constructing the vector with 3–4 separate packaging plasmids, with minimal overlapping sequences between and within them^31^. The preference for intragenic integration of LV vectors, coupled with the SIN design considerably reduces their genotoxicity potential; Indeed, recent LV clinical trials with a follow up of nearly 10 years have shown no genotoxicity resulting from LV vectors, even though, preclinical studies have reported vector integration in known oncogenes (MLL, NUP214)^32^ and suggested that transcriptionally active enhancers like the LCR can lead to gene dysregulation, independent of the vector type (RV or LV) or design (LTR-based or SIN)^26,33^.

Our group explored the genotoxicity potential of LCR enhancer elements and showed that the LCR-containing LVs have approximately 200-fold lower immortalization potential than RVs. Though gene dysregulation was seen in the vicinity of the integrated vector, no proto-oncogene upregulation was noted^34^. Use of cHS4 insulators decreased the transforming potential further, along with decreasing position dependent variable gene expression and methylation-associated silencing^35^.

## Preclinical studies

### Gene Therapy for β-Thalassemia

LV-mediated human β-gene transfer was shown to rescue mouse models of β-thalassemia intermedia and β-thalassemia major. May and colleagues demonstrated the use of a LV vector carrying the human β-globin gene fragment and β-globin LCR spanning the HS2, HS3, and HS4 regions to correct thalassemia intermedia in mice with increase in hemoglobin levels by 3–4 g/dl^36^. The same group developed an adult β0-thalassemia major mouse model using mice engrafted with beta-globin-null Hbb(th3/th3) fetal liver cells and rescued their severe phenotype using the same vector with an average vector copy number (VCN) of 1.0– 2.4^18^. Imren and coworkers thereafter showed correction of β-thalassemia mice using a vector carrying the βT87Q gene, where a point mutation in the β-globin gene also confers it with anti-sickling properties. However, multiple copies were required for adequate correction of the mouse thalassemia phenotype^32^. Our group showed complete correction of the human β0 thalassemia phenotype in vitro and in a xenograft model with approximately 2 vector copies/cell^37^. Miccio and colleagues used a LV vector carrying the β-globin gene linked to a minimized LCR HS2/HS3. They showed that a frequency of 30–50% of transduced hematopoietic cells harboring an average VCN of 1 was sufficient to fully correct the thalassemia phenotype in th3/+ mice^15^. In addition they also demonstrated that the genetically corrected erythroblasts had an *in vivo* survival advantage, thus encouraging the need to explore the utility of reduced intensity transplant regimens for clinical gene therapy trials.

### Gene Therapy for SCD

The efficacy of LV-mediated transfer of γ-globin gene/mutated β-globin genes (βT87Q and βAS3) for correcting SCD was explored using transgenic and humanized xenograft sickle cell murine models. Pawliuk and colleagues showed improvement in hematological parameters, splenomegaly and hyposthenuria in BERK and SAD mice using the βT87Q LV^38^. Levasseur on the other hand used a βAS3 (a human β-globin gene with 3 anti-sickling mutations) in a LV to successfully transduce murine HSC without cytokine stimulation^39^. Romero et al used the same βAS3 LV to successfully transduce bone marrow CD34 progenitor cells from patients with SCD, and produce sufficient levels of anti-sickling hemoglobin^40^. Persons et al^41^, and Pestina et al^42^ showed improvement in SCD phenotype by increasing the expression of fetal hemoglobin (HbF) using γ-globin LV. Our group showed an 18–20% engraftment of HSCs containing γ-globin gene-LV, following a non-myeloablative conditioning regimen. This donor chimerism was sufficient to result in approximately 60% circulating RBC containing HbF (F cells) with an improvement in the SCD manifestations^43^.

## Clinical trials

The success in preclinical models, supported by safety studies on LV vectors led to the design of clinical gene therapy trials. Cavazzana-Calvo et al. enrolled a hemoglobin E/β (βE/β0)-thalassemia major patient in 2007 who received genetically modified autologous HSCs expressing βT87Q-globin following myeloablative busulfan conditioning. This subject became transfusion independent 1–2 years later^44^ with a hemoglobin maintained at 9–10 gm/dl. This therapeutic benefit was initially due to a clonal expansion observed following vector insertion in the HMGA2 gene. However this clone has eventually subsided. The trial was subsequently extended to include 18 patients with thalassemia (transfusion-dependent βEβ0 n=10, β0β0 n=5, β+thalassemia n= 3) and 4 patients with SCA^45–47^. All patients with βEβ0-thalassemia and β+thalassemia became transfusion independent within a year of transplant, with a median increase in hemoglobin by 4.9-g/dl, while patients with β0β0-thalassemia with a similar hemoglobin increase experienced a significant reduction in their transfusion requirement, but were not transfusion independent, since the baseline hemoglobin levels in β0β0thalassemia are much lower than in individuals with β+/βE thalassemia. One of four patients with SCD who received a high dose of transduced CD34 cells had remarkable improvement in their SCD phenotype.

Two other trials are using the β-globin LV vectors for β-thalassemia. In the trial led by Boulad et al (NCT01639690), the preconditioning regimen had to be switched from a reduced-intensity busulfan to myeloablative doses following modest engraftment and β-globin expression with the lower dose^48^; the trial led by Ferrari et al (NCT02453477) is using a myeloablative regimen consisting of treosulfan and thiotepa with initial success^48^. The NCT02247843 trial led by Kohn et al. for SCD is investigating the efficacy of βAS3LV, and the trial led by our group (Malik et al.; NCT02186418) is using a γ-globin LV following reduced intensity conditioning. The results of these studies are eagerly awaited.

## Recent Advances in Genetic Manipulation Technology

The emergence of gene editing technology, which enables precise genome manipulation, offers a new approach for treating β-hemoglobinopathies^49^. Site specific double strand breaks (DSB) can be induced with zinc finger nucleases, transcription activator-like effector nucleases (TALENS), meganucleases and more recently with Clustered regularly interspaced short palindromic repeats (CRISPR)/Cas9 system. CRISPR/Cas9 has revolutionized gene targeting. Unlike other nucleases which use a protein dimer for target sequence recognition and require a novel protein to be engineered for each new target site, CRISPR/Cas9 technology uses a short guide RNA (gRNA) with a 20bp sequence complementary to the DNA sequence to be targeted^50^. In addition targeting/knockdown of multiple genes can be achieved by using multiple gRNAs with a common Cas9 protein^51^. DSB is then followed by DNA repair through one of the two major pathways: 1) Non-homologous end joining (NHEJ) with direct fusion of the nuclease cleaved ends. This repair mechanism is error-prone, leading to indels, and is cell-cycle stage independent^52^. 2) Homologous directed repair (HDR) uses an exogenous donor template^53^ delivered via single-stranded oligonucleotides, plasmids, or viral vectors like integrase deficient lentivirus or adeno-associated virus^54^, for gene correction with targeted insertion. For hemoglobinopathies, gene editing strategizes shown to be successful include either induction of endogenous fetal hemoglobin^55,56^, modification of the causal β-globin gene mutation by targeted nucleases^57^ or therapeutic transgene integration^58^, or a combined approach^59,60^. Inactivation of an erythroid specific enhancer of BCL11A by gene editing leads to suppression of BCL11A and up-regulation of γ-globin in erythroid lineage cells^61,62^.

These gene editing strategizes are being performed in CD34+ stem and progenitor cells^63^ or in induced pluripotent stem cells (iPSCs) capable of differentiating into any somatic cell type^64–66^. Patient-specific iPSCs are generated by the genetic reprogramming of their somatic cells, and provide an unlimited source of stem cells which can be genetically manipulated, differentiated along a specific tissue type and returned back to the patient. Currently, active research in differentiating iPSC towards definitive hematopoietic stem cells with long term engraftment potential is underway. In addition to their therapeutic potential, iPSCs can also be used as *in-vitro* disease models^67^. Off-target nuclease binding activity^68^, efficient means of delivering the genome editing tools to target stem cell populations without loss of ‘stemness’, genomic variation occurring with somatic reprogramming, efficient gene targeting by homology directed repair^69^, and developing functional HSCs from these genetically modified iPSC^70,71^ are some of the challenges in the field.

## Conclusions and future directions

Gene therapy for hemoglobinopathies is now a reality, with several patients cured of their β0/βE thalassemia or with significant amelioration from β0/β0 thalassemia and one patient with SCD, while others are showing modest transgene expression. The current curative capacity of gene transfer technology is limited by the severity of the underlying disease. The increase in hemoglobin to 8–9 gm/dl seen in β0β0 thalassemia is still not sufficient to prevent ineffective erythropoiesis, and hence these subjects are still intermittently transfused. However the overall transfusion burden in this patient population has decreased dramatically. Cohen et al showed that the success of chelation therapy in achieving a neutral or negative iron balance (assessed by liver iron concentration) had a significant correlation to the transfusion iron intake^72^. Thus decreasing the transfusion burden is advantageous, as it not only might affect the dose of chelation therapy used but also affects its outcome.

The challenges to efficacious clinical translation in hemoglobinopathies include the dose of engraftable transduced HSCs, the intensity of the preconditioning transplant regimen, and expression of the transgene. In-vivo selection strategies can ensure expansion of the few genetically-modified engrafted HSCs. Improving vector potency will augment gene expression. Efforts to promote differentiation of iPSC technology to produce engraftable HSC can expand the HSC source, and gene editing can circumvent the need for high transgene expressing-LV and potential, albeit low, insertional genotoxicities of LV. New technologies that can reshape the future of gene therapy are gene editing using CRISPR/CaS9 and development of hematopoietic stem cells from iPSCs with long term repopulating potential, although much work is needed to make this a reality. With scientific advancements in stem cell biology and genetic manipulation, we envision a future where a child prenatally diagnosed with hemoglobinopathy can have his/her genetically modified cord blood stem cells transfused even before the fetal to adult hemoglobin switch, thus preventing the occurrence of any disease manifestations.
